# Design and production of a fish feed pelletizing machine

**DOI:** 10.1016/j.heliyon.2019.e02001

**Published:** 2019-06-24

**Authors:** Paul Chukwulozie Okolie, Iheoma Chigoziri Chukwujike, Jeremiah Lekwuwa Chukwuneke, Jude Ezechi Dara

**Affiliations:** a Mechanical Engineering Department, Nnamdi Azikiwe University, PMB 5025, Awka, Anambra State, Nigeria; b Polymer and Textile Engineering Department, Nnamdi Azikiwe University, PMB 5025, Awka, Anambra State, Nigeria

**Keywords:** Mechanical engineering, Fish feed, Homogenous, Production, Farmers, Pelletizer, Mixture

## Abstract

Animal feeds contributes to a greater percentage of the cost of production of livestock. For the increment in productivity and profit, farmers are advised to produce their feed themselves to reduce the cost of production. This work is aimed at producing a simple single unit fish feed pelletizer at a lost cost for peasant farmers. A fish feed pelletizer has been designed and constructed. It consists of hopper, screw conveyor, barrel, dies, drives system and heater. The design was carried out using engineering principles with due consideration to cost, ease of operation, serviceability, durability, and performance. It is designed to be driven by a 1.5 HP, three-phase electric motor with a heating element of 1500 W attached to the barrel surface to ensure adequate heating of the feed as they travel through the barrel. The test that determines the performance of the pelletizer was carried out which showed a throughput capacity of 17 kg/h, machine efficiency of 73.33% and a pelletizing efficiency of 90.90% with low mechanical damage of 9.10%. The cylindrical pellets size produced by the pelletizer was in the range of 2–8 mm diameter, which is suitable for fish and poultry farming. The machine was produced using locally sourced materials at a production cost of one hundred and eight thousand naira only (N 108,000.00).

## 1 Introduction

An artificial feed is important for the improvement of fisheries and achieving maximum yields from resources of fresh water (Bhosale et al., 2010). Fish meal is considered to be the best ingredients, due to its compatibility with the protein requirement of fish (Alexis et al., 1996). Augusto et al. (1973), Fagbenro (1988), Kwari and Igwebuike (2001), Diarra et al. (2001) and along with other researchers, opined that the utilization of various forms of farm and agro-industrial wastes and by-products for the formulation of complete feeds for livestock, poultry and aquatic life is feasible. The production of feed for livestock, poultry and aquatic life involves series of activities such as grinding, mixing, pelleting and drying operations (Balami et al., 2013). Investigation shows that the few available small-scale processing equipment are not very efficient, thus increasing the inability of farming activities. Historically, the pellet has been used by various industries to describe a variety of agglomerates produced from different and diverse raw materials. The proper use of traditional methods in existence and modern development derived from various researches have really assisted in animal feed production through the processing of raw food items. Furthermore, the investigation carried out by Amadi (2007) has shown that a pelleting machine is capable of creating cylindrical pellet from a blend of dry powdered stocks, such as molasses, steam or as a machine that is used to convert materials and food items such as maize, groundnuts and millets with other additives in good ratio.

The process of extrusion is used to produce Pellets. According to Mc-Donald et al. (1995), earlier, the feeding of fish, poultry and other animals was based on local methods characterised by the milling of grains and cereals as meals on mortar and stones. The safe and healthy food preparations for the animals could not be attained let alone the trend of growth and development, with the aim of achieving aesthetic and market needs of these animals. Research show that animals preferably fed on solid and soft nutritious meals, and the pelleting machine is one of the equipment that can be used to prepare this mixture of nutrients in powdered form.

Further, it was stated by Kumar (1992) that pelleting machines can serve the following functions: (1) moulding of feed meals like soft capsules for animals, which can be eaten easily by fish and poultry animals (2) production of sawdust that can be used in a pellet stove (3) production of iron ore pellets that varies in diameters for blast pig iron production (4) production of chemical pellet. Moreover, the design and construction of an electrically motorised operated pelleting machine are expedient in order to improve food security and technology for the comfort to animals such as fish, turkey, chicken, birds, etc. Hence this work is geared towards producing a simple single unit pelletizer for small scale farming. The pelletizer should be capable of reducing the moisture content of the pellets, thereby reducing the stress that accompanies sun drying in small scale feed production.

## 2 Materials and method

### 2.1 Machine description

The important components of the pelleting machine are the hopper where the feed meal is fed into the machine and the pelleting chamber in the form of worm auger or screw shaft which is seen to propel the feed. The shaft is operated by pulley and belt system that is powered by an electric motor.

**Fig. 1:**
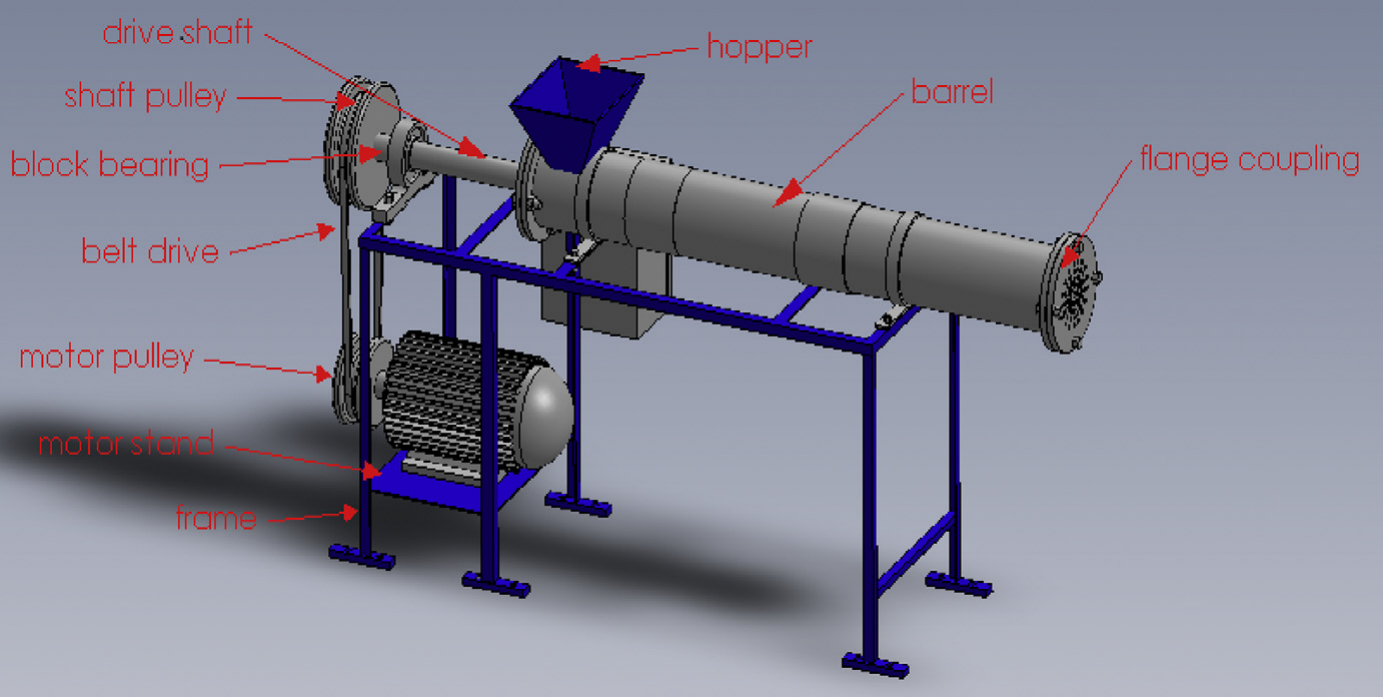
3D drawing of the fish feed pelletizer.

**Fig. 2:**
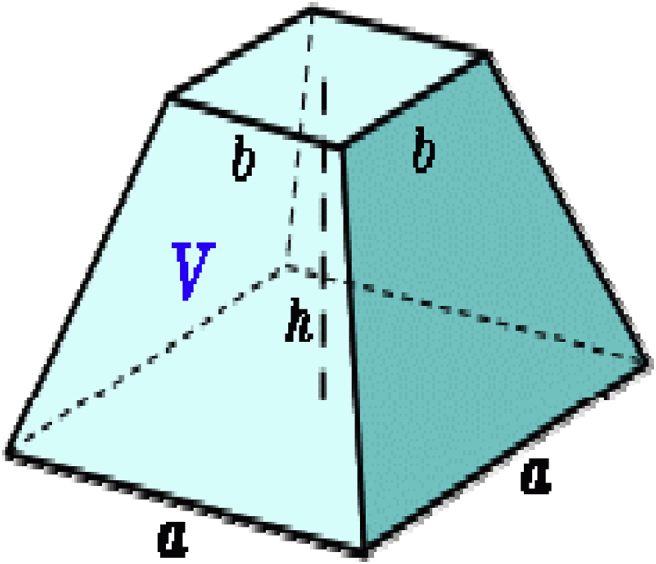
Hopper.

**Fig. 3:**
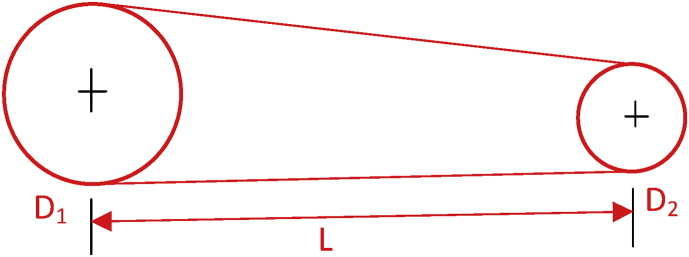
Schematic diagram for belt and pulley design.

The output pellet is produced by compacting and forcing through a die opening via a mechanical process (see Figs. 1–3).

### 2.2 Design requirements

For the design of pelleting machine, Mott (1985) asserted that the general requirements are:

• To steadily receive the mixture of feed into the machine.
• To introduce the mixture of feeds into the cutting unit uniformly
• To uniformly cut the mixture of feeds
• To steadily discharge the pellets out of the machine with ease.

### 2.3 Material selection and production planning

In this work good material selection was undertaken to achieve the following purposes: convenient maintenance, to reduce the noise of the machinery, to produce the machinery at an economical cost, to improve and reduce dimensions, to achieve machine aesthetics, to improve the strength of the machinery, to improve its resistance to wear, corrosion and corrosive medium (Sharma and Aggar-Wal, 1998; Chukwulozie et al., 2015). The materials utilised for the construction of the machines are; mild steel, stainless steel, cast iron, and chemical paint.

### 2.4 Machine design

#### 2.4.1 Hopper design

The shape of the hopper is in the form of a truncated pyramid with an inclination angle of 60° to enable conveying and storage of raw materials.

(1) $$\text{Volume}\;\text{of}\;\text{hopper}\;=\left({\;\text{a}}^{2}+{\text{b}}^{2}+\text{ab}\right)\text{h}/3$$

Whereb=150mm,a=450mm,h=350mmtherefore,V=[4502+1502+(150×450)]3503=34125000mm3$\text{V}\;=\;0.0341{\text{m}}^{3}$

The mass flow through the hopper using Johanson equation is calculated thus:

(2) $$\text{Mass}\;\text{flow}={\text{ρ}}^{0}\text{A}\sqrt{\frac{\text{Bg}}{2\left(1+\text{m}\right)\tan\emptyset }}$$

$\text{Where}:{\;\text{ρ}}^{0}\;=\;\text{bulk}\;\text{density}\;\text{of}\;\text{the}\;\text{material}\;\left(1300\text{kg}/{\text{m}}^{3}\text{assumed}\right)$$\text{A}=\;\text{surface}\;\text{area}\;\text{of}\;\text{hopper}\left(\;\text{W}\;\text{x}\;\text{L}=\;0.45\;\text{x}\;0.45=\;0.2025{\text{m}}^{2}\right)$Ø=hopperangle$\text{g}=\;\text{acceleration}\;\text{due}\;\text{to}\;\text{gravity}\left(9.807\;\text{m}/{\text{s}}^{2}\right)$m=0(symmetricalslothopper)B=w=0.45m

$$\text{therefore}\;\text{mass}\;\text{flow}\;\text{through}\;\text{the}\;\text{hopper}=1300\times 0.2025\sqrt{\frac{0.45\times 9.807}{2\;\tan\;45}}$$

=306.99996kg/s

#### 2.4.2 Capacity design for a given electric motor

The design for motor output power enables appropriate selection of a motor with enough power to start and run the machine at full load.

(3) Power=F×V

WhereP=PowerinwattsF=RotationalforceactingontheshaftinNewton(N)v=linearvelocityoftheshaft(m/s)

(4) ButF=ma

wherem=Massofrotatingshaftinkilogram(kg)$\;\text{a}=\text{Angular}\;\text{acceleration}\;\text{of}\;\text{the}\;\text{motor}\;\text{in}\;\text{radian}/\text{seconds}\;\text{square}\;\left(\text{rad}/{\text{s}}^{2}\right)\;$

(5) also,a=ω²r

ω=angularvelocityofthemotorinradian/secondsω=v/r

(6) Thereforev=ωr

By putting Eq. (5) into (4)

(7) F=mωr

By putting Eqs. (7) and (6) into (3)

(8) P=mω²r×ωr

(9) Butω=2πN/60

Putting (9) into (8)

(10) $${\text{P}=8\text{m}\left(\frac{\text{πN}}{60}\right)}^{3}{\text{r}}^{2}$$

#### 2.4.3 Determination of screw conveyor diameter and power to drive conveyor

The diameter and power of the screw conveyor required for conveying material at a rate of 20 kg/h for the capacity of a continuous screw conveyors were calculated from the expression given by (Spivakovsky and Dyachkov, 1967).

(11) $${\text{D}}^{2}=\frac{4\text{Q}}{60\text{π}\left(\text{Snφpc}\right)}$$

Where,Q=capacityofscrewconveyorS=screwpitchn=speedofconvey=loadingefficiencyp=freebulkdensityofthematerial,c=loadingfactordependingontheinclinedangletothehorizontal

The recommended values by (Spivakovsky and Dyachkov, 1967) for slow flowing abrasive material are

$$\text{S}\;=\;0.8\text{D},\;\;\text{φ}\;=\;0.125\;\text{and}\;\text{c}\;=\;1{\;\text{for}\;\text{inclination}\;\text{angle}\;\text{b}}_{0}\;=\;0.\;$$

The recommended minimum and maximum speed of conveyor are 200–490 rpm (Spivakovsky and Dyachkov, 1967)

$${\text{D}}^{3}=\;4\;\times \;20/60\;\times \;3.142\;\times \;0.8\;\times \;0.125\;\times \;475\;\times \;785\;$$

D=0.105m=105mm

(12) $$\text{Pr}\;=\;\text{QL}\left({\text{ω}}_{0}+\text{sinβ}{}^{\circ}\right)/367$$

ωois4.0forslow–flowingabrasivematerial, ${\text{inclination}\;\text{angle}\;\text{of}\;\text{conveyor}\;\text{β}}^{\text{o}}\;\text{is}\;0^{\text{o}}\text{and}\;$

L=lengthofconveyor

Pr=9×20(4+0)/367

Pr=1.96kw

#### 2.4.4 Length of screw

This is determined from the length to diameter ratio (L_s_: D) of the screw. It is the ratio of the flight length of the screw to the original diameter. A ratio of 13:1 was selected for portability. This means that the flight length of the screw is 13D (where D = original diameter of the screw).

The feed section, transition section and the metering section are in the ratios of 4D:4D:5D respectively.

Feedsectionlength(Fl)=4D=4×105=420mm

Feedsectiondepth(Fd)=0.2D=105×0.2=21mm

Transitionsectionlength(Tl)=4D=4×105=420mm

Meteringsectionlength(Ml)=5D=5×105=525mm

Meteringsectiondepth(Md)=0.33Fd=0.33×21=6.93mm

For standard screw profile, the angle the flight makes with a line perpendicular to the shaft

$\;\left(\text{Flight}\;\text{angle}\right)\;\text{is}\;17.6568^{\text{o}}.$

(13) Thereforethepitchofthescrew,S=π×D×tanδ

Where;D=diameterofscrewð=flightangleFlightwidth(screwthickness)=0.1D=10.5mmScrewbarrelclearance=0.17D=17.85mm

#### 2.4.5 Design of drive system (Belt and pulley design)

The machine runs with a 1400 rpm motor which will produce a speed reduction of 70 rpm. This reduces the speed of the motor via a V – belt before it enters the shaft. The smaller pulley is adapted at the motor and connected to the bigger pulley on the shaft of the screw via a belt drive. The bigger pulley welded to the shaft of the screw which passes through two pillow bearing.

#### 2.4.6 Determination of pulley diameter

The speed of driving pulley versus speed of driven pulley can be expressed by Shigley and Mischike (2001) and Khurmi and Gupta (2006) as;

(14) $${\text{D}}_{1}{\text{N}}_{1}={\;\text{D}}_{2}{\text{N}}_{2}$$

Where, ${\text{D}}_{1}$ and${\text{N}}_{1}$are the diameter and speed of driving pulley.

Also, ${\text{D}}_{2}$ and ${\text{N}}_{2}$are the diameter and speed of driven pulley.

In other to get a speed of 112 rpm on the drive shaft using an electric motor of 1400 rpm, the diameter of the driven pulley is calculated thus using a pulley of 40 mm diameter for the driver;

$$1400\text{rpm}\times \;40\text{mm}\;=\;112\text{rpm}\times {\;\text{D}}_{2}$$

$${\;\text{D}}_{2}\;=\;1400\;\text{x}\;40/70$$

$${\;\text{D}}_{2}=\;500\text{mm}$$

Therefore, for a driving pulley of 40 mm diameter, the driven pulley diameter was calculated from the above as 500 mm.

#### 2.4.7 Determination of belt length

With known pitch diameters of pulleys D_2_ = 500 mm, D_1_ = 40 mm and center distance between motor/shaft pulley, C = 340 mm. The length of belt required is calculated using the equation below:

(15) $$\text{L}\;=\;2\text{C}\;+\;\left({\;\text{D}}_{2}-{\text{D}}_{1}/4\text{C}\right)\;+\;1.57\left({\;\text{D}}_{2}\;+{\;\;\text{D}}_{1}\right)$$

L=2(340)+[500−40/(4×340)]+1.57(500+40)

L=2027.77mm

#### 2.4.8 Determination of tension in the belt

Tension T_1_ acting on the tight side of the belt and the tension T_2_ acting on the slack side of the belt. The values of T_1_ and T_2_ are calculated using the Eq. (16);

(16) $$\frac{{\text{T}}_{1}-{\text{mv}}^{2}}{{\text{T}}_{2}-{\text{mv}}^{2}}=\;\text{e}\frac{\text{f}\phi }{\text{sin}5.0\text{θ}}$$

Where; m = mass of a unit h of the belt

v = Linear velocity of belt;
ϕ = Angle of wrap on pulley (rad)
μ = The friction coefficient between belt & pulley.
T_1_ = Tension in the tight side of belt (N)
T_2_ = Tension in the slack side of (N)
θ = Groove angle for v – belt (degree).

The maximum tension in the tight side of the belt depends on the allowable stress of the material. For a B section belt the following parameters are given:

T_1_ = allowable tension = 900 N,
θ = 38 ± 0.50P, f = 0.2

(17) $$\;\text{The}\;\text{linear}\;\text{velocity}\;\text{is}\;\text{given}\;\text{by}\;\text{V}\;=\;\frac{\text{πDN}}{60}$$

V=3.142x0.04x1400/60

V=2.93m/s

For small pulley

$$\phi \;=\;\phi_{1}=\;161.7{}^{\circ}\;\left(2.82\;\text{rad}\right)\;\text{using}\;\text{θ}\;=38.5{}^{\circ}\;\left(0.672\;\text{rad}\right)\;$$

e×μ×α/sin5.0θ=5.54

Forlargepulley,ϕ=ϕ₂=198.26°(3.46rad)

e×μ×α/sin0.5θ=8.61

The small pulley with the highest value of 5.54 will be used as a basis for the design. Substituting the value of T_1_ = 900 N, m = 0.19 and V into the equation. The tension T_2_ on the slack side can be calculated as:

$${{900\;–\;0.19\left(2.93\right)}^{2}/{\text{T}}_{2}\;–\;0.19\left(2.93\right)}^{2}=\;5.54$$

$${\text{T}}_{2}=\;\left(898.4\;+\;9.03\right)/5.54$$

$${\text{T}}_{2}=163.8\text{N}$$

#### 2.4.9 Design of the barrel

The barrel is designed to withstand a high temperature up to 400 °C and to be a very good conductor of heat. The barrel is made of stainless steel which has a melting point of 1515 °C and a thermal conductivity of 50.2 W/m^°^C.

(18) $$\text{Volume}\;\text{of}\;\text{barrel}\;=\;\text{volume}\;\text{of}\;\text{cylinder}\;={\;\;\text{πr}}^{2}\text{h}$$

$${=\;\left(114\text{mm}\right)}^{2}\;\text{x}\;3.142\;\text{x}\;860\text{mm}$$

$$=\;35116751.52{\text{mm}}^{3}$$

#### 2.4.10 Design of the frame

The base is designed to withstand the torque generated by the electric motor.

The torque generated is calculated thus;

(19) Torque=9.549P/N(engineeringtoolbox.com)

Where; P = electric motor horse powers

N = number of revolutions

Torque=9.549×2/1400=0.00670Nm

The frame is designed using mild steel angel iron of tensile strength of 290 N/mm^2^ (Khurmi and Gupta, 2006). The list of the input and output parameters are shown in Table 1, below.

**Table 1:** The table showing list of input and output parameters.

INPUT PARAMETERS			OUTPUT PARAMETERS		
Parameters	Symbol	Unit	Parameters	Symbol	Unit
Acceleration due to gravity	G	m/sec^2^	Angular velocity of the motor	Ω	rad/sec
Angle of wrap	Α	rad	Screw barrel clearance	Sbc	Mm
Bulk density	*ρ*^o^	kg/m^3^	Angular acceleration of the motor	Α	rad/sec^2^
Capacity of screw conveyor	Q	kg/hr	Feed section length	Fl	Mm
Coefficient of friction between the belt and the pulley	Μ	-	Length of belt	L	Mm
Convey speed	N	rpm	Power required to drive the conveyor	Pr	kw
Diameter of driven pulley	D_2_	mm	Flight width	Fw	Mm
Diameter of driving pulley	D_1_	mm	Metering section depth	Md	Mm
Flight angle	Δ	degree	Feed section depth	Fd	Mm
Groove angle	Θ	degree	Tension on the tight and slack side of the belt	T_1_ and T_2_	N
Hopper angle		degree	Volume of hopper	V	m^3^
Hopper height	H	mm	Rotational force acting on the shaft	F	N
Hopper inlet width	A	mm	Mass flow rate	M	kg/s
Hopper outlet width	B	mm	Power of the electric motor	P	kw
Loading factor	C	-	Screw length	L_s_	Mm
Radius of the shaft	R	mm	Screw diameter	D	Mm
Rotational speed of shaft	N	Rpm	Screw pitch	S	Mm
Speed of the electric motor	N_1_	Rpm	Transition section length	Tl	Mm
Speed of driven pulley	N_2_	Rpm	Metering section length	Ml	Mm

### 2.5 Machine construction

In the construction of the machine, a lot of parameters were considered.

#### 2.5.1 Barrel construction

The barrel is constructed by machining a round hollow mild steel pipe into an appropriate length of 860 mm. It has an outer diameter of 114 mm and an inner diameter of 106 mm with a thickness of about 4 mm. Circular wedges with holes for the passing of bolts were attached to the barrel at both ends. This was done for the ease of maintenance and assembly of the inner components (i.e. screw and bearing replacement and adjustment). A circular-shaped heating element of 2000 W 415 V and a thermocouple was fixed on the barrel surface to ensure thermo-mechanical heating of the resin pellets and thermal regulation.

#### 2.5.2 Screw and shaft construction

A 114 mm screw conveyor was constructed by a normal machining process. It was reduced to 105 mm making a clearance of 4.5 mm between screw and barrel. The shaft protrudes out of the casing along the drive section of the machine and entered the bearing housing before it finally enters the larger pulley. The shaft also extends out of the die plate centre and the second bearing is housed inside the barrel. A bearing of suitable dimension was also purchased for use in the extruder. The shaft is 1190 mm in length and 35 mm in diameter.

#### 2.5.3 Die design and construction

The extruder needed a die which could sustain the high pressure of material conveyed by the feed screw. The die was made using 8 mm thickness mild steel plate. It has a diameter of 204 mm and is removable. Finally, sixty (60) numbers of holes were drilled around the surface of the die plate to make way for the resin pellets to pass through. The diameter of each drilled hole is 4 mm.

#### 2.5.4 Machine construction

**Fig. 4:**
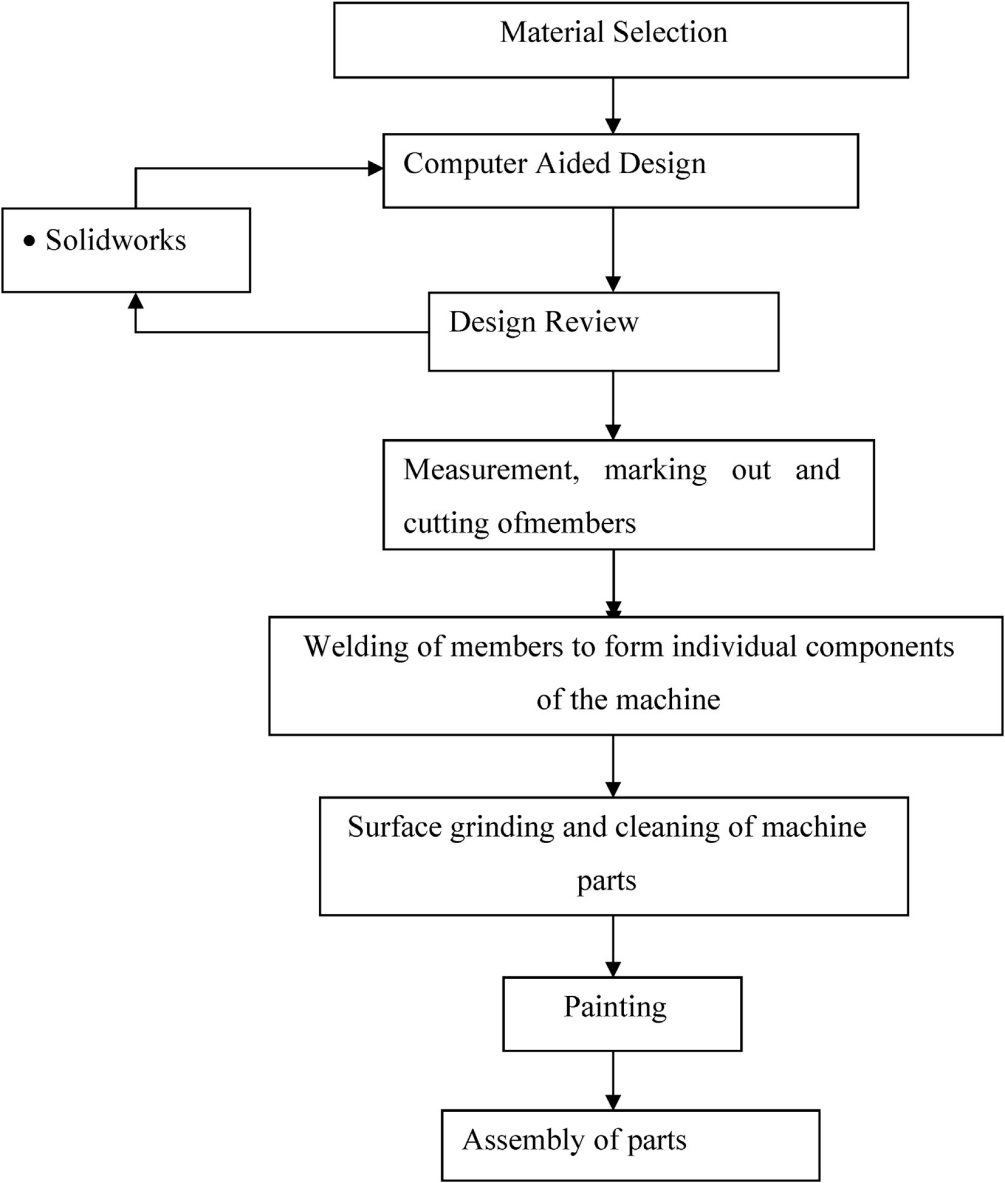
Flowchart of design steps.

**Fig. 5:**
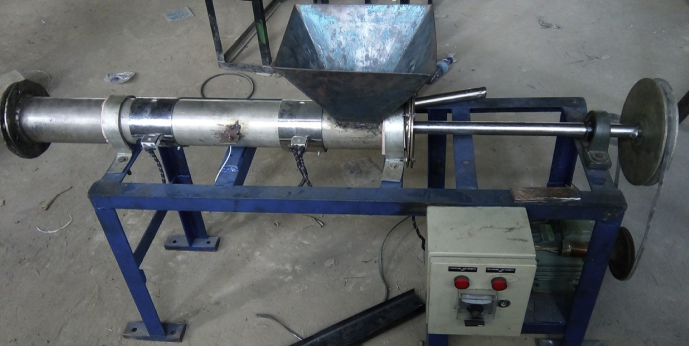
Fish feed pelletizer picture.

**Fig. 6:**
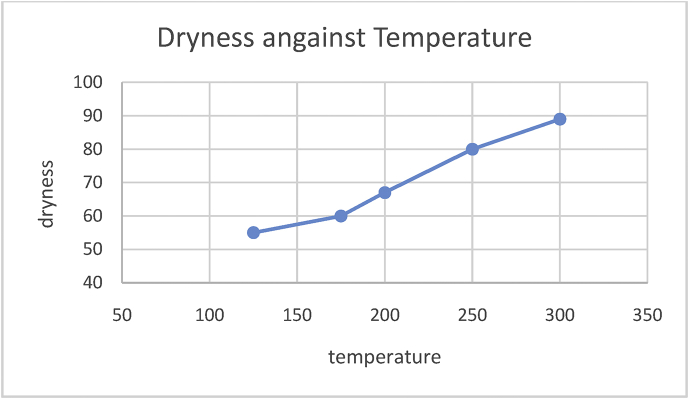
Graph of dryness against temperature.

**Fig. 7:**
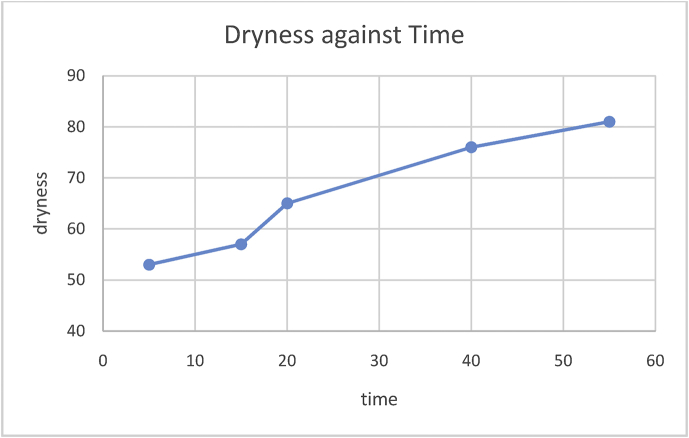
Graph of dryness against time.

The machine was fabricated based on the design specification. The construction was carried out with locally sourced materials to reduce the cost of production to meet the design objective. Each of the components was designed and fabricated following the due fabrication process as shown in Fig. 4. This entails marking and cutting out the required shape and dimension, welding of the parts to form the components, and surface finishing to improve on the aesthetics. The fabricated components were then assembled to give the desired machine shown in Fig. 5 below (see Figs. 6 and 7).

#### 2.5.5 Bill of material selection and cost

The following were put into consideration in selecting the materials for the construction of palm nut and fibre separator;

i. Availability of materials
ii. Suitability of the materials for effective operation
iii. Cost effectiveness.

Table 2 below shows the machine components, the materials selected for the components, and the cost.

**Table 2:** The table showing the cost of the machine.

S/N	MATERIALS	SPECIFICATION	QTY	UNIT PRICE (N)	TOTAL (N)
1	Frame	2″ angular bar 3 mm thick	2 length	3,500	7,000
2	Hopper	Sheet metal 1800 mm × 400 mm x 2 mm	1 sheet	2,000	2,000
3	Conveyor shaft	45 mm thick stainless pipe,	1500 mm	10	15,000
4	Barrel	4″ stainless pipe	1000 mm	15	15,000
5	Electric hearter	415 V Heater	2 num	3,000	6,000
6	Control board	Control pannel	1 num	7,500	7,500
4	Electric motor	3-phase 2 hp	1 num	25,000	25,00
5	Bearing	45 mm bearing	2 num	4,500	9,000
6	Pulleys	500 mm, 40 mm	1 num	3,000	3,000
7	Belt	Type A v-belt	1 num	1,000	1,000
8	Electrode	Guage 10	½ packet	5,000	2,500
8	Cutters	Big cutter	1	1,500	1,500
9	Paint		1 tin	1,500	1,500
10	Transport				2,000
11	Labour				10,000
	**Total**				**108,000**

Hence the total cost for the production of the pelletizing machine is one hundred and eight thousand naira (N 108,000), which is equivalent to three hundred and eight dollars ($ 308).

### 2.6 Performance tests

A series of test was carried out after the design and construction of the machine, to determine the throughput capacity, machine efficiency, pelletizing efficiency and dryness of the feed. The homogeneous feed was weighed and fed into the pelletizer to obtain the dry pelletized feed. The time taken for complete pelletizing was taken, the pelletized sample was weighed, and the dry feed was collected for the test. The throughput capacity, machine efficiency, and pelletizing efficiency was calculated using Eqs. (20), (21), and (22) below.

The throughput capacity (Tpc) is given as;

(20) $$\text{Tpc}=\;\frac{{\text{W}}_{\text{R}}}{\text{t}}$$

Where W_R_ is the recovered weight after pelletizing, and t is the time taken for complete pelletizing.

The machine efficiency (ε) is given as:

(21) $$\text{ε}=\frac{{\text{W}}_{\text{R}}}{{\text{W}}_{\text{F}}}$$

Where W_F_ is the weight of feed fed into the machine

Pelletizing efficiency (ɳ) is given as;

(22) $$\text{η}=\frac{{\text{W}}_{\text{P}}}{{\text{W}}_{\text{R}}}$$

Where W_P_is the weight of the pelletized sample.

#### 2.6.1 Throughput capacity

The throughput is the rate at which the feed sample fed into the machine is been recovered. This was obtained using Eq. (20). In this work, 3 kg of the homogenous feed mixture was fed into the machine and 2.2 kg of the feed was recovered at a time interval of 8 min. This gave a throughput capacity of 0.275 kg/min, which is approximately 17 kg/h.

#### 2.6.2 Machine efficiency

The machine efficiency is the ratio of the weight of the feed sample fed into the machine to the weight of the feed sample recovered after the pelletizing process. This was calculated using Eq. (21), which gave a machine efficiency of 73.33%. Hence the percentage loss in the pelletizing process is 26.67%. This loss is significant as a result of the clearance between the tip of the screw and the barrel.

#### 2.6.3 Pelletizing efficiency

The pelletizing efficiency was obtained using Eq. (22). This was used to determine the effectiveness of the machine in producing the pellets. The ratio of the weight of the pellets to the weight of the recovered feed gives the pelletizing efficiency. The weight of the pellets (W_P_) was determined by weighing the pellets, which was manually separated from the recovered feed. The separated pellets weighed 2.0 kg. Hence the separating efficiency was obtained to be 90.90%. However, the mechanical damage obtained was 9.10% which arose from poor kneading as the feed conveys along the barrel.

#### 2.6.4 Feed dryness test

Feed dryness test was conducted by varying the temperature of the heater on the barrel (at a constant time) and the moisture of the feed was determined. Also, at a constant temperature, time is varied to know the effect of it on the feed. The results of the test are shown below (see Tables 3 and 4):

**Table 3:** Dryness of feed at a varying temperature.

S/N	1	2	3	4	5
TEMPERATURE (°C)	125	175	200	250	300
DRYNESS (%)	55	60	67	80	89

**Table 4:** Dryness of feed at a varying time.

S/N	1	2	3	4	5
TIME (seconds)	5	15	20	40	55
DRYNESS (%)	53	57	65	76	81

From the above graphs, it is shown that as the temperature of the heater increases the rate of moisture removal from the feed increases. Also, as time increases the moisture removal increases.

## 3 Conclusion

This work successfully designed and produced a simple single fish feed pelletizing machine for peasant farmers. Effective design and adequate material selection criteria were employed in the course of production of the machine. It was designed to be driven by a 1.5 HP, three phase electric motor with a heating element of 1500 W attached to the barrel to eliminate the problem of sun drying that is associated with available local pelletizers. The performance test was carried out to determine the throughput capacity, machine efficiency and pelletizing efficiency which gave a value of 17 kg/h, 73.33% and 90.90% respectively. However, low mechanical damage of 9.10% was obtained. The cylindrical pellets size produced by the pelletizer was in the range of 2–8 mm diameter, depending on the size of die used for the pelletizing. Therefore the machine is suitable for small and medium scale fish and poultry farming. The machine was produced using locally sourced materials at a production cost of one hundred and eight thousand naira only (N 108,000.00) which is relatively cheap compared with available local pelletizers without a heating system.

## Declarations

### Author contribution statement

Okolie Paul Chukwulozie, ChukwujikeIheoma Chigoziri, Chukwuneke Jeremiah Lekwuwa & Dara Jude Ezechi: Conceived and designed the experiments; Performed the experiments; Analyzed and interpreted the data; Contributed reagents, materials, analysis tools or data; Wrote the paper.

### Funding statement

This work was supported by the Tertiary Education Trust Fund (TETfund), through Institution Based Research grant (IBR). Years 2012–2014 Merged TETFUND Research Projects (RP) Intervention Funds 8th Batch. (2017).

### Competing interest statement

The authors declare no conflict of interest.

### Additional information

No additional information is available for this paper.
